# Prevalence and knowledge of medical students about the risks of E-cigarette use

**DOI:** 10.5588/ijtldopen.24.0642

**Published:** 2025-06-13

**Authors:** M.A. Souza de Castro, G.F. Machado, M.M. Zamboni, P. Normando, M.B. Conte

**Affiliations:** Faculdade de Medicina de Petropolis/UNIFASE (Petropolis School of Medicine; PSM), Brazil.

**Keywords:** tobacco, Brazil, medical students, lung injury, smoking prevalence

Dear Editor,

Tobacco consumption remains a significant global health issue, contributing to morbidity and mortality. According to the WHO 2021 Global Report on Tobacco Trends (2000–2025), more than 8 million deaths in 2019 were attributed to tobacco-related diseases, including cardiovascular and respiratory illnesses, different types of cancer and other debilitating conditions.^1^ Electronic nicotine delivery systems (ENDS) and electronic non-nicotine delivery systems (ENNDS), collectively referred to as e-cigarettes, are battery-powered devices that aerosolize nicotine or psychoactive substances. Promoted as safer alternatives to traditional tobacco products and as smoking cessation tools, these devices have gained popularity, particularly among younger populations.^2,3^ However, several authors have already demonstrated an association between e-cigarette use and significant health risks, including cardiovascular disease, nonspecific respiratory diseases and e-cigarette-induced lung injury (EVALI).^4–6^ Health professionals play a key role in reducing tobacco consumption by serving as role models for health practices and promoting smoking cessation, particularly when they abstain from smoking themselves.^7^ Medical students, as future physicians, represent a key target for tobacco prevention programs due to their potential influence on societal smoking behaviors. In this sense, global research emphasized the importance of understanding their smoking habits and knowledge to enhance their contributions to public health efforts.^8,9^

Given the increasing experimentation with and awareness of e-cigarettes, particularly among younger individuals, and the limited data on e-cigarette use among Brazilian medical students, this study aimed to assess the prevalence of e-cigarette use and evaluate their knowledge about e-cigarettes and associated health risks in one private school of Medicine located in the Rio de Janeiro State (Brazil).

We conducted a cross-sectional study using an 11-item online survey supported by REDCap (Research Electronic Data Capture), a secure web application for building and managing online surveys and databases. The survey consisted of seven core questions addressing smoking prevalence, knowledge of e-cigarette components, and associated health risks, and it was accessible for 14 days. To ensure consistency, we defined the prevalence of both e-cigarettes and conventional cigarette current smoking as any use within the past 30 days and the prevalence of prior experience as any use more than 30 days ago, rather than adopting conventional cigarette definitions of the medical literature. Participants were eligible if they were medical students aged 18 years or older, actively enrolled from the 1^st^ to 6^th^ years in a school of Medicine, with internet access, and had provided electronic consent. Students who did not complete all core questions were excluded from the analysis. The target sample size was calculated to ensure a 5% margin of error at a 95% confidence level, assuming a 15% prevalence of e-cigarette current smoking or prior experience, resulting in a required sample size of 196 participants. Assuming a 20% response rate among 1,100 eligible students, a minimum of 220 responses was anticipated. Ethical approval was obtained from the Institutional Review Board of the Petropolis School of Medicine.

Among the 275 students enrolled in the study, 89.4% (246/275) responded to all core questions regarding the prevalence of prior experience and current e-cigarette smoking, while 99.3% (273/275) responded to all core questions in the knowledge assessment, with mean age respectively of 23.3 years (±SD 6.10) and 23.28 years (±SD 5.81). The survey results are summarized in the Table. The prevalence of prior experience (52.4%; 129/246) and current e-cigarette use (18.6%; 46/246) in our study is consistent with a multinational study conducted between 2020–2021,^10^ which reported prior experience and current use rates of 44% and 20% in Brazil (n=3,093) and 47% and 11% in the USA (n=3,097), whereas India (n=1,366) exhibited a markedly lower prevalence (<1%). Our findings are also similar to a survey of medical students in the United Kingdom (2019–2020), which found that 32.6% (n=188) reported current or past e-cigarette use.^11^ A systematic review assessing the prevalence of e-cigarette use among medical students in Saudi Arabia, including studies from 2010–2020, reported rates ranging from 10.6–27.7%.^12^ Conversely, Martins et al. (2023) reported lower prevalence among Brazilian medical students (n=700) from various medical schools (2016–2018), with 13.1% having experimented and only 2.3% reporting current use.^13^ Bertoni et al. (2021) and the National Institute of Cancer Brazil estimate that the prevalence of e-cigarette use among the general Brazilian population, aged 18 years or older, ranges from 0.64% in 2019 to 2.1% in 2023.^14,15^ The discrepancies observed between findings for the general population in Brazil and those reported by Martins et al. (2023), Degani-Costa et al. (2022), and our study among medical students, as well as those from other countries, warrant further investigation. Our study was conducted at a private medical school, and Martins et al. (2023) identified an association between e-cigarette experimentation and enrollment in private institutions (adjusted OR = 3.83; 95% CI, 2.00–7.36). Additionally, a general observation of recent studies is a higher prevalence of e-cigarette use.

**Table.** Responses to a survey on prevalence and knowledge about e-cigarettes. All results expressed as % responding Yes n/total prevalence responses included.

			Enrolled year in Medical School^A^					
		Total	1^st^ year	2^nd^ year	3^rd^ year	4^th^ year	5^th^ year	6^th^ year
Prior experience with conventional smoking and/or e-cigarettes	% Yes	55,7%	37.1%	53.5%	57.7%	64.4%	58.8%	56.75%
		(137/246)	(13/35)	(15/28)	(30/52)	(38/59)	(20/34)	(21/37)
Prior experience with e-cigarettes	% Yes	52.4%	37.1%	46.4%	53.8%	59.3%	58.8%	51.4%
		(129/246)	(13/35)	(13/28)	(28/52)	(35/59)	(20 /34)	(19/37)
Current e-cigarettes smoke	% Yes	18.6%	17.1%	17.9%	11.5%	25.4%	23.5%	13.5%
		(46/246)	(6/35)	(5/28)	(6/52)	(15/59)	(8/34)	(5/37)
Knowledge of components of e-cigarettes smoke	% Yes	82.1%	86.8%	81.8%	78.6%	82.5%	90.2%	73.2%
		(224/273)	(33/38)	(27/33)	(44/56)	(52/63)	(37/41)	(30/41)
Knowledge about cardiovascular risk	% Yes	94.5%	94.7%	90.9%	91.1%	98.4%	92.7%	100.0%
		(258/273)	(36/38)	(30/33)	(51/56)	(61/62)	(38/41)	(41/41)
Knowledge about nonspecific respiratory risks	% Yes	97.8%	92.1%	100.0%	96.4%	100.0%	100.0%	97.6%
		(267/273)	(35/38)	(33/33)	(54/56)	(63/63)	(41/41)	(40/41)
Knowledge about EVALI risk	% Yes	23.1%	23.7%	12.1%	10.7%	33.3%	39.0%	17.1%
		(63/273)	(9/38)	(4/33)	(6/56)	(21/63)	(16/41)	(7/41)

A Missing = 1 case due to unfulfillment of year enrolled in the Medical Course

EVALI = Electronic cigarette-induced lung injury; n = number.

Although 97.8% (267/273) of students were aware of nonspecific or general respiratory risks associated with e-cigarette smoking, only 23.1% (63/273) were familiar with EVALI. We found no statistically significant differences in the prevalence of e-cigarette use, knowledge of e-cigarette components, or awareness of EVALI risks between students in the early years (1st and 2^nd^) and later years (5^th^ and 6^th^) of their medical education, both in the general sample and sex and age groups (p < 0.05).

Medical students, as future healthcare professionals, should have a thorough understanding of the risks associated with all forms of smoking, including emerging alternatives like e-cigarettes, to effectively counsel patients and advocate for tobacco control. This study highlights the need for targeted educational strategies to address e-cigarette use and its health risks among Brazilian medical students. The findings suggest critical gaps exist in knowledge, risk perception and regulatory understanding, which has significant implications for educators, policymakers and healthcare professionals. By highlighting these deficiencies, we aim to promote discussion on the impact of e-cigarette consumption among medical students, contributing to stronger health advocacy and policy development. Moreover, integrating electronic cigarettes – alongside conventional tobacco smoking – into medical curricula is essential, not merely as a risk variable but as a critical public health issue, ensuring that future physicians are adequately prepared for their roles in public health. Despite standardized National Curriculum Directives in all medical schools in Brazil (both private and public), the prioritization of education on e-cigarette and tobacco smoking remains unclear. Further studies with a broader cohort of medical students are necessary to confirm these findings and inform possible curricular enhancements.

## References

[1] WHO global report on trends in prevalence of tobacco use 2000–2025, fourth edition. Geneva: World Health Organization; 2021.

[2] U.S. Department of Health and Human Services. E-Cigarette Use Among Youth and Young Adults. A Report of the Surgeon General. Atlanta, GA: U.S. Department of Health and Human Services, Centers for Disease Control and Prevention, National Center for Chronic Disease Prevention and Health Promotion, Office on Smoking and Health, 2016.

[3] Cao DJ, Review of Health Consequences of Electronic Cigarettes and the Outbreak of Electronic Cigarette, or Vaping, Product Use-Associated Lung Injury. J of Med Toxicology 2020;16:295-310. 10.1007/s13181-020-00772-w PMC7320089 32301069

[4] Alzahrani T, Association between electronic cigarette use and myocardial infarction. Am J Prev Med 2018;55(4):455–61. 30166079 10.1016/j.amepre.2018.05.004 PMC6208321

[5] McConnell R, Electronic cigarette use and respiratory symptoms in adolescents. Am J Respir Crit Care Med. 2017;195(8):1043–9. 27806211 10.1164/rccm.201604-0804OC PMC5422647

[6] Werner AK, Hospitalizations and Deaths Associated with EVALI, N Engl J Med 2020;382(17):1589-1598. 32320569 10.1056/NEJMoa1915314 PMC8826745

[7] Frank E, Predictors of physician’s smoking cessation advice. JAMA 1991;266:3139-3144. 1956100

[8] Richmond R. Teaching medical students about tobacco. Thorax 1999;54:70–78. 10343637 10.1136/thx.54.1.70 PMC1745356

[9] Harrabi I, Medical students and tobacco in 2004: a survey in Sousse, Tunisia. Int J Tuberc Lung Dis. 2006;10(3):328-32. 16562715

[10] Degani-Costa LH, Vaping and Hookah Use Among Medical Trainees: A Multinational Survey Study. Am J Prev Med 2023;65(5):940-949. 37178979 10.1016/j.amepre.2023.05.009

[11] Afzal M, Electronic cigarette use and perceptions amongst UK medical students: A cross-sectional study. Tob Prev Cessat 2021;7:13. 33644495 10.18332/tpc/131826 PMC7894361

[12] Patil S Prevalence of electronic cigarette usage among medical students in Saudi Arabia - A systematic review. Niger J Clin Pract 2022;25(6):765-772. 35708416 10.4103/njcp.njcp_2006_21

[13] Martins SR, Prevalence and associated factors of experimentation with and current use of water pipes and electronic cigarettes among medical students: a multicentric study in Brazil. J Bras Pneumol. 2023;49(1):e20210467. 36700569 10.36416/1806-3756/e20210467 PMC9970372

[14] INCa. Instituto Nacional de Câncer. (Brazilian National Institute of Cancer). Dispositivos Eletrônicos para Fumar (DEF; Electronic Nicotine Delivery Systems; ENDS). Statistical data on the prevalence of the use of ENDS. National Institute of Cancer Brazil https://www.gov.br/inca/pt-br/assuntos/gestor-e-profissional-de-saude/observatorio-da-politica-nacional-de-controle-do-tabaco/dados-e-numeros-do-tabagismo/def-dados-e-numeros

[15] Bertoni N, Prevalence of electronic nicotine delivery system sand water pipe use in Brazil: where are we going? Rev Bras Epidemiol 2021;24(suppl2):e210007. 10.1590/1980-549720210007.supl.2 34910061

